# Contemporary climate change and terrestrial invertebrates: evolutionary versus plastic changes

**DOI:** 10.1111/eva.12116

**Published:** 2013-11-06

**Authors:** Menno Schilthuizen, Vanessa Kellermann

**Affiliations:** 1 Naturalis Biodiversity CenterLeiden, The Netherlands; 2 Centre for Ecological and Evolutionary Studies, Rijksuniversiteit GroningenGroningen, The Netherlands; 3 Institute Biology Leiden, Leiden UniversityLeiden, The Netherlands; 4 School of Biological Sciences, Monash UniversityClayton, Vic., Australia

**Keywords:** adaptation, *Drosophila*, insects, land snails, phenotypic plasticity, temperature stress

## Abstract

To forecast the responses of species to future climate change, an understanding of the ability of species to adapt to long-term shifts in temperature is crucial. We present a review on evolutionary adaptation and phenotypic plasticity of temperature-related traits in terrestrial invertebrates. The evidence for adaptive evolution in melanization is good, but we caution that genetic determination needs to be tested in each individual species, and complex genetic correlations may exist. For phenological traits allochronic data sets provide powerful means to track climate-induced changes; however, rarely are responses deconstructed into evolutionary and plastic responses. Laboratory studies suggest climate change responses in these traits will be driven by both. For stress resistance, the evidence for shifts in traits is poor. Studies leaning heavily on *Drosophila* have demonstrated potential limits to evolutionary responses in desiccation and heat resistance. Quantifying the capacity for these species to respond plastically and extending this work to other taxa will be an important next step. We also note that, although not strictly speaking a species trait, the response of endosymbionts to heat stress requires further study. Finally, while clearly genetic, and possibly adaptive, the anonymous nature of latitudinal shifts in clines of genetic markers in *Drosophila* prevents further interpretation.

## Introduction

Climate is one of the most important factors that shape the evolution of species' niches (Mayr 1963; MacArthur 1972). Most species live within a relatively narrow portion of the world's available climatic breadth. Yet, because of climate unpredictability, species have evolved a certain amount of tolerance to various abiotic climate-related factors in their responses to warming and cooling events. Such responses may have adapted due to natural selection, in which case genetic traits that provide a higher fitness under the new climate regime are selected, or they may be phenotypically plastic, where the organism can adjust its phenotype without any genotypic change (West-Eberhard 2003). Given the climate warming of the past few decades, interest is mounting in disentangling the evolutionary and plastic components of species' responses to climate change (Gienapp et al. 2008). Forecasting climate-warming-associated changes in geographical distribution, population density, habitat choice, and trophic interactions of species, all of which are relevant for assessing the likelihood of population survival under climate change scenarios (but see Kopp and Hendry 2014 for caveats), requires an understanding of the degree of evolvability and plasticity in a broad range of species' traits.

Merilä and Hendry (2014) point out that temporal changes in climate-related traits of wild species, were, until recently, often interpreted as adaptive evolution, without firm evidence that the alternative (namely phenotypic plasticity) can be excluded. On the other hand, the demonstration of phenotypic plasticity itself also requires firm evidence. They provide a framework for a critical approach to this problem, based on the premise that neither alternative (adaptive evolution nor phenotypic plasticity) is a ‘null’ model: both require positive evidence. Therefore, empirical studies that claim one or the other process should be supported by a strict evaluation of the necessary information, that is, evidence for a genetic basis, for natural selection having operated, and for nonplasticity of the trait in question, in the case of adaptive evolution. Conversely, if phenotypic plasticity is proffered as an explanation, then evidence for a plastic response in the trait must be accompanied by evidence that the trait is not genetically determined and has not undergone natural selection. Moreover, both processes could be operating at the same time and the relative contributions of both must be assessed (Merilä and Hendry 2014). In climate change studies, such criteria are particularly hard to meet, because these are usually allochronic in approach and cannot benefit from the many advantages (Endler 1986) of synchronic studies.

In this paper, we evaluate published evidence for adaptive and plastic changes in climate-related traits in terrestrial invertebrates. For different sets of species, some traits may be more or less important in terms of climate change responses. For terrestrial invertebrates whose distributions are governed largely by climate (Addo-Bediako et al. 2000; Angilletta 2009), the traits that are likely to matter include the innate stress tolerances of a species, which are often used as proxies for susceptibility to climate change (Deutsch et al. 2008; Huey et al. 2009; Sunday et al. 2011; Kellermann et al. 2012b) and the ability to track optimal environments via behavioural thermoregulation (Kearney et al. 2009b; Huey et al. 2012). Both will depend on the mobility/dispersal ability of a species. For species with high dispersal ability, it may be possible to track optimal conditions (which will often result in latitudinal shifts). However, for species with low dispersal, novel ‘solutions’ (either genetic or plastic) may arise to combat stressful environments, for example, the degree of melanization linked to thermoregulation (Majerus 1998), as well as phenological traits such as emergence time, generation time and diapause. At the same time, such solutions may generate ecological mismatches (Donnelly et al. 2012). Finally, climate change will also impact species interactions. A prime example is the potential for the ecological tolerances of obligate endosymbiotic bacteria to dictate species responses to climate change (Ohtaka and Ishikawa 1991; Wernegreen 2012). For many of these traits, the lack of sufficient data sets limits our ability to assess current responses to changing climates. Here, we highlight what we have learned from field and laboratory-based studies.

### Dispersal and selection of habitats

One way in which terrestrial invertebrates may deal with high external temperatures is by behavioural thermoregulation, that is, actively selecting sites in the environment that fall within their temperature tolerance range, and avoiding those that do not. Adaptive changes in behavioural thermoregulation, however, would depend on (i) the presence of a heterogeneous environment, (ii) the evolvability of microhabitat preference and/or dispersal-related traits (Huey et al. 2012).

For species occupying homogeneous environments such as the understory of tropical rainforests or deserts with little canopy cover, the availability of microclimates is likely to be reduced. For example, cactus roots are unlikely to offer any form of refuge for the highly heat-resistant cactophilic desert *Drosophila* species (Gibbs et al. 2003) and thus heat resistance in desert *Drosophila* species tracks maximum temperature of the environment much more closely than in nondesert *Drosophila* species (Kellermann et al. 2012a,b). Dispersal ability likewise may be a limiting factor for some terrestrial invertebrates. While highly mobile flying insects may be able to actively move through their environment to select thermally suitable microsites, more sessile species such as land snails or flatworms are much more handicapped in this respect.

For species that are faced with a thermally heterogeneous habitat, climate change may select for changes in behavioural thermoregulation. Such changes are expected to be visible especially at the leading or trailing edges of a distributional range, where the species will often experience many conditions at the lower or upper end of its tolerance range. In the butterfly *Aricia agestis*, Thomas et al. (2001) documented a decrease in avoidance of a host plant in the UK at the leading edge of its expanding range. The host plant was associated with hot microhabitats, and the avoidance behaviour was shown to be genetic. Similarly, climate change may select for dispersal ability at the leading range edge. The same paper reports that, over the last few decades, the proportions of long-winged morphs in the bush crickets *Conocephalus discolor* and *Metrioptera roeselii* had increased in an area of the UK where this species was in the process of colonizing northward. Wing length in these species is phenotypically plastic (Zera and Denno 1997), and, although the authors interpret the change as evolutionary adaptation, they report only circumstantial evidence for a genetic component; as far as we are aware, a genetic basis has not yet been confirmed. Similarly, Hill et al. (1999) report in the skipper butterfly, *Hesperia comma*, genetically based investment in thoracic (flight) muscles to be greatest in distant, newly colonized patches of habitat.

### Melanization

The biophysical properties of an organism's external surface form an important target for evolutionary adaptation or plastic response to a changed environment. Changes in colour, texture and composition of skin, carapace or shell can modify heat exchange across the interface between the organism's internal and external environment (Trullas et al. 2007). By far the most studies have been devoted to colour polymorphisms. In terrestrial invertebrates, these have always been a favourite trait system for ecological geneticists, with industrial melanism in *Biston betularia* as the prime example (Brakefield 1987). In general, good evidence exists for the genetic basis of skin or carapace pigmentation in many species (e.g. Wittkopp et al. 2009), and the biophysical effects are often (but by no means always–see below) straightforward.

Several studies have made use of the well-studied shell colour polymorphism in the European land snail *Cepaea nemoralis*. These polymorphisms involve the shell ground colour (which ranges from pale yellow to deep brown) and the number and widths of dark brown spiral bands. The classical genetics of this colour polymorphism is well studied, and most colour morphs can be traced to the expression of a limited number of Mendelian genes, usually with full dominance (Lang 1904; Cain et al. 1960; Cook 1967; Murray 1975). Plasticity has not been reported in the expression of any of these loci, but a limited plastic component may be suspected in the expression of spiral bandwidth (R. Cameron, personal communication). Field experiments on individual plasticity to test this are ongoing (M. Schilthuizen & L. den Daas, unpublished data).

Biophysical experiments have shown that lighter *Cepaea* shells allow the soft body inside to maintain a lower body temperature than darker shells (Heath 1975; Steigen 1979), and field studies support this (Richardson 1974; Jones et al. 1977). In addition, data exist that the snails' activity patterns are also linked with shell colour, with darker snails seeking out more humid and shaded positions, and lighter snails remaining active for longer under dry conditions (Jones et al. 1977; Jones 1982; Kavaliers 1992; Ozgo and Kubea 2005). Shell colour allele frequency differences in different habitats are in the direction expected through thermal selection, with snails markedly lighter in open habitats than in adjacent, shaded habitats (Cain and Sheppard 1950; Jones et al. 1977; Silvertown et al. 2011; Schilthuizen 2013). Allele shifts take place very rapidly after experimental (Ozgo and Bogucki 2011) or natural (Ozgo 2011; Schilthuizen 2013) colonization of novel habitat patches. Although predator-induced selection for cryptic coloration may confound the thermally selected patterns, under conditions where these confounding effects can be excluded, allele frequency patterns are as predicted under thermal selection (Jones et al. 1977; Ozgo and Kinnison 2008; Ozgo 2011; Silvertown et al. 2011).

Several allochronic studies of *Cepaea nemoralis* populations that span the past 45 years or so have shown frequency increases of alleles that code for lighter shells (Ozgo & Schilthuizen 2012; Cameron et al. 2013), sometimes only in more exposed habitats (Silvertown et al. 2011; Cameron and Cook 2013), and these changes thus can be linked to climate warming imposing a selection coefficient on these alleles of the order of a few percent (Cameron and Cook 2013). Several studies (e.g. Ozgo & Schilthuizen 2012; Cameron and Cook 2013) show that the frequency increases in alleles for light shells were accompanied by an overall increase in habitat shading. Because shaded habitat selects for darker shells, both under thermal and predator selection, this reinforces the interpretation that overall climate warming is the driver for these changes.

Overall, the evidence for evolutionary adaptation of shell colour to climate in *Cepaea nemoralis* is quite strong. The genetic basis of the traits is firmly established, and plastic responses are likely to be only minimally involved. However, the system is complex, with historical and genetic contingencies and several sometimes opposing selective agents operating simultaneously. Selection by thermal maxima is often hard to isolate as a single factor (Jones et al. 1977; Ozgo 2008).

Conversely, in *C. hortensis*, there are indications that selection by temperature minima, rather than maxima is crucial in certain aspects of shell colour change. In hibernating *C. hortensis*, pale yellow shells are better protected against severe winter colds, although the biophysical and/or physiological mechanisms for this are not understood (Jones et al. 1977; Cain 1983; Cameron and Pokryszko 2008; R. A. D. Cameron, personal communication). A population in England was resurveyed repeatedly, showing the frequency of alleles for yellow and loss of bands had declined between the mid-1960s and 2007, in correspondence with fewer wintertime extreme cold spells (Cameron and Pokryszko 2008).

**Figure 1:**
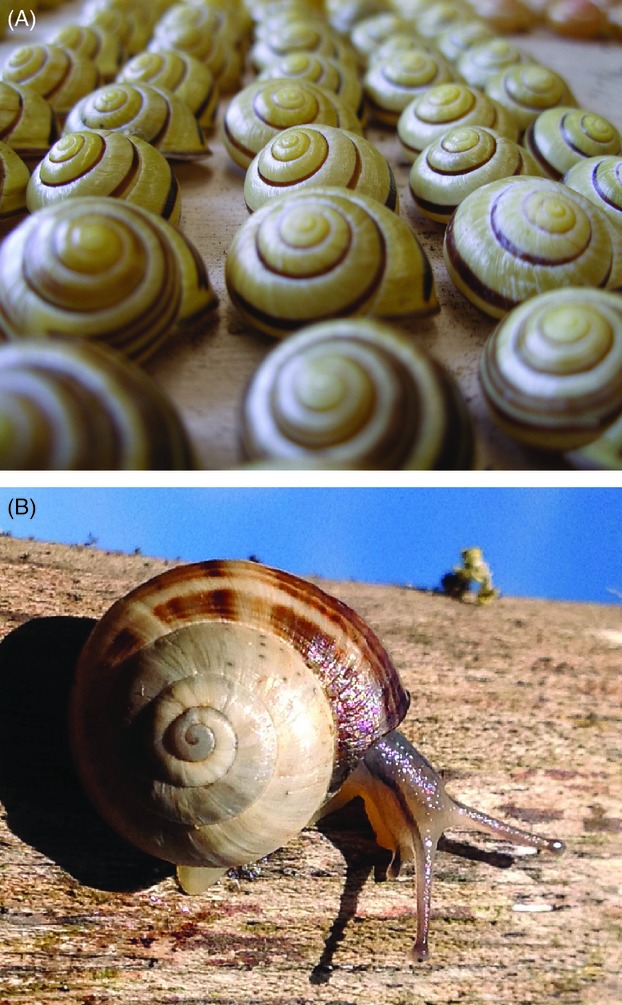
Melanization patterns in land snails may be genetically determined or phenotypically plastic. (A) shows *Cepaea nemoralis* shells of the colour morphs Y12345 (in the foreground) and Y00300 (in the background), respectively, of genotype *C*^*Y*^*C*^*Y*^*B*^*B*^*B*^*B*^*U*^*5*^*U*^*5*^ and *C*^*Y*^*C*^*Y*^*B*^*B*^*B*^*B*^*U*^*5*^*U*^*3*^ *C*^*Y*^*C*^*Y*^*B*^*B*^*B*^*B*^*U*^*3*^*U*^*3*^ (Murray 1975). (B), on the other hand, shows a *Theba pisana* shell (like *C. nemoralis*, belonging to the Helicidae), in which the expression of banding pattern has changed during ontogeny—which may indicate a degree of phenotypic plasticity for banding expression in this species (after Köhler et al. 2013).

The *Cepaea* studies have spurred work on thermal selection on shell coloration in other terrestrial gastropods, such as *Theba pisana* (Fig. 1). Like in *Cepaea*, shell colour pattern in this latter species varies from light to dark and at least three loci are involved (Cowie 1984). Several studies, including allochronic ones, have found correlations between high temperatures and the prevalence of light-coloured morphs in juvenile and adult snails (e.g. Johnson 1980, 2011, 2012). However, unlike in *Cepaea*, there are indications of phenotypic plasticity, with modification in the expression of, for example, bandwidth and intensity, during ontogeny, as well as an association between shell colour and the inducibility of heat-shock proteins (Köhler et al. 2009, 2013). Moreover, the role of shell coloration in influencing a snail's body temperature has been called into question (Scheil et al. 2012).

In addition to the coloration of the shell, the degree of pigmentation of the snail skin, for which the genetic basis has been confirmed in *Cepaea* (Murray 1975), but not in *Theba*, has also been shown to correlate negatively with average temperature in several land snail studies (Cowie and Jones 1985; Cowie 1990). Like in land snail shells, the colour-based heat absorbance of the external skeleton has also been linked with environmental temperature in several species of insects. In the ladybird beetle, *Adalia bipunctata*, which, like many members of this family, show very conspicuous variation in the colour patterns on the elytra and pronotum, colour polymorphism is genetic (Majerus 1994, 1998). Melanic and nonmelanic morphs in this species have different fitness curves at different temperature regimes (Brakefield and Willmer 1985; de Jong et al. 1996), and clines exist that suggest temperature-induced selective responses of melanization alleles (Majerus 1994), which have been shown to shift in response to climate change (Brakefield and de Jong 2011). Similarly, in the pygmy grasshopper, *Tetrix undulata*, distinct associations are seen between (presumably genetically determined) body colour and behavioural response to temperature (Forsman et al. 2002 and references therein). In *Drosophila melanogaster*, melanization clines exist on different continents (Munjal et al. 1997), with expression changes in candidate genes linked to plasticity in this trait (Telonis-Scott et al. 2011).

However, these examples should not be taken to imply that all colour polymorphisms that correlate with environmental temperature are evidence for adaptation. Many cases of phenotypically plastic cuticle coloration are known in insects (Karlsson and Forsman 2010; Mitchie et al. 2010; and references therein), even in species closely related to those for which genetic colour polymorphism is uncontested. In fact, we suspect that phenotypic plasticity may play a more important role in the latter category of cases than anticipated. For example, the genetic basis for colour polymorphism in *Tetrix undulata* has not been demonstrated directly. Rather it has been inferred from related species where this is the case. This may, however, be risky, as even closely related species (and even conspecific populations—Husby et al. 2010) may differ in the degree by which genes control colour phenotype.

Even the adaptive significance of body colour under different thermal regimes may not be as straightforward as assumed. Several studies have shown differences in activity pattern between light- and dark-coloured morphs (Wittkopp and Beldade 2009), for example in snails (Wolda 1965) and pygmy grasshoppers (Forsman et al. 2002). The latter case may be particularly illustrative: experiments suggest that, rather than a direct effect of body colour, the behavioural differences are the result of genetic correlations, and colour polymorphism is part of a genetic complex for alternative strategies for dealing with temperature. The complexity of this system makes it hard to predict the fitness effects of temperature change. Moreover, melanin may also play an important role in immunity, thus trade-offs and pleiotropic effects could be expected (Wittkopp and Beldade 2009).

### Life-history traits

Shifts in traits linked to climate change have been demonstrated for a number of phenological traits in insects. These have been reviewed extensively in Donnelly et al. (2012), but briefly, earlier emergence, changes in generation times, coupled with an increase in number of generations per year, timing of migration and an increase in period of activity are just some of the phenological traits that have shifted over the last 150 years (see Table 1 for references). Common garden experiments describing population differentiation and geographical clines for traits such as development time, voltinism and reproductive diapause suggest that genetic variation underlies these traits (Griffiths et al. 2005; Karl et al. 2008; Bentz et al. 2011; Valimaki et al. 2013). Phenological traits, however, also tend to be highly dependent on the thermal environment, displaying a high level of phenotypic plasticity (Tauber et al. 1986; Bradford and Roff 1995; Nylin and Gotthard 1998; Bentz et al. 2011).

**Table 1:** Summary of studies on terrestrial invertebrates implicitly or explicitly designed to examine plastic and/or genetic responses of traits driven by climate change

Higher Taxon	Species	Trait_type	Genetic	Plastic	Adapt	Cause	Driver	Time?	Reference(s)
Lepidoptera	*Hesperia comma*	DH	.	.	.	Y(2)	DD	FD	Thomas et al. (2001)
Lepidoptera	*Hesperia comma*	DH	Y(2)	N(2)	.	Y(2)	DD	MD	Hill et al. (1999)
Lepidoptera	*Aricia agestis*	DH	Y(2)	N(2)	.	Y(2)	DD	FD	Thomas et al. (2001)
Orthoptera	*Conocephalus discolor*	DH	.	.	.	Y(2)	DD	FD	Thomas et al. (2001)
Orthoptera	*Conocephalus discolor*	DH	.	.	.	Y(2)	DD	FD	Thomas et al. (2001)
Orthoptera	*Tetrix undulata*	DH	Y(2)	N(2)	N(2)	.	TP	.	Forsman et al. (2002)
Coleoptera	*Adalia bipunctata*	ME	Y(2)	N(2)	Y(2,3)	Y(2)	DD	EX,FD	Majerus (1994, 1998), Brakefield and Willmer (1985), de Jong et al. (1996), Brakefield and de Jong (2011)
Pulmonata	*Cepaea nemoralis*	ME	Y(2)	N(2)	Y(2)	Y(2)	TP	EX,FD	e.g., Murray (1975), Heath (1975), Jones et al. (1977), Silvertown et al. (2011), Ozgo & Schilthuizen (2012); Cameron et al. (2013)
Pulmonata	*Cepaea hortensis*	ME	Y(2)	N(2)	Y(2)	Y(2)	TP	FD	Cameron and Pokryszko (2008)
Pulmonata	*Theba pisana*	ME	Y(2)	N(2)?	Y(2)	Y(2)	TP	FD	Johnson (1980, 2011, 2012), Cowie (1984) Scheil et al. (2012)
Lepidoptera	Butterfly and moth species	LH	.	.	.	Y(2)	TP	FD	Roy and Sparks (2000), Altermatt (2010), West-wood and Blair (2010), Poyry et al. (2011)
Lepidoptera	*Lobesia botrana*	LH	.	.	.	Y(2)	TP	FD	Martin-Vertedor et al. 2010;
Hymenoptera	*Apis mellifera*	LH	.	.	.	Y(2)	TP	FD	Sparks et al. (2010)
Insecta	14 insect species	LH	.	.	.	Y(2)	TP, PR	FD	Ellwood et al. (2012)
Odonata	Dragonfly species	LH	.	.	.	Y(2)	TP	FD	Hassall et al. (2007), Dingemanse and Kalkman (2008), Doi (2008)
Hemiptera	Aphid species	LH	.	.	.	Y(2)	TP, PR	FD	Zhou et al. (1995), Harrington et al. (2007)
Diptera	*Wyeomyia smithii*	LH	Y(1,2)	.	.	Y(2)	NS	FD	Bradshaw and Holzapfel (2001)
Diptera	*Drosophila melanogaster*	AN	Y(6)	.	.	Y(1)	NS	FD	Anderson et al. (2005), Umina et al. (2005)
Diptera	*Drosophila subobscura*	AN	Y(6)	.	.	Y(1)	TP	FD	Balanya et al. (2006)
Diptera	*Drosophila robusta*	AN	Y(6)	.	.	Y(1)	TP	FD	Etges and Levitan (2008)

Trait_type (type of trait examined): DH = traits influencing dispersal and habitat selection, ME = melanisation, LH = phenology and other life-history traits, TD = thermal and drought stress tolerance, SY = symbionts, AN = anonymous genetic traits. A ‘Y’ indicates that evidence was found for genetic or plastic responses in traits or that adaptability or causality was investigated; ‘N’ indicates evidence was not found; ‘.’ indicates that it was not investigated; '?' indicates that there is some controversy over a particular issue. Numbers next to a ‘Y’ or ‘N’ denote the method of investigation invoked, in cases with no numbers, a method was invoked that does not fit into one of the categories used for this review. Genetic categories: 1 = animal models, 2 = common garden studies, 6 = molecular genetic approaches; plastic categories: 2 = common garden studies; adapt categories: 2 = phenotypic selection estimates, 3 = genotypic selection estimates; cause categories: 2 = phenotype by environment interactions. For full descriptions of all categories, see Merilä and Hendry (2014). Primary driver (causal driver of change): NS = not specific, TP = temperature, PR = precipitation, DD = dispersal distance; Time? (time component included in data collection): EX = field or greenhouse experiment through time, FD = field observations through time, MD = modelled through time.

Extensive allochronic data sets have demonstrated shifts in these traits but rarely do these studies go beyond associating changes in traits with climate. With both evidence for genetic variation and plasticity underlying these traits, the relative contribution of genetics versus plasticity remains to be determined. One of the few examples that has demonstrated a genetic shift in response to climate is that of reproductive diapause in the pitcher plant mosquito *Wyeomyia smithii* (Bradshaw and Holzapfel 2001). The reproductive diapause phenotype, shown to have a genetic basis, reduced in frequency correlating with an increase in temperature over a 30-year period.

### Thermal and drought stress tolerance

A species innate stress tolerance is likely to be a key in mediating climate change responses in ectotherms (Angilletta 2009). Measures of thermal tolerance and stress resistance have robustly been linked to latitudinal/environmental data both within and between species (Parkash and Munjal 1999; Addo-Bediako et al. 2000; Hoffmann et al. 2002; Kellermann et al. 2012a) and are often used as proxies for climate change risk (Deutsch et al. 2008)— that is, ‘space for time substitutions’ (Merilä and Hendry 2014). Large-scale studies pooling data on upper and lower thermal limits are rapidly accumulating (Deutsch et al. 2008; Huey et al. 2009; Sunday et al. 2011; Kellermann et al. 2012b), but as static single point estimates, often compiled from numerous studies, they provide no current means for tracking temporal changes. Nevertheless, consistent associations between temperature, latitude and stress resistance across a wide range of terrestrial invertebrates are indicative of adaptive processes underlying these traits.

For terrestrial invertebrates, temporal data sets of thermal tolerances and stress resistance simply do not exist. For *Drosophila*, one of the best-studied systems with respect to thermal traits, studies have examined patterns of heat, cold and desiccation resistance across latitude and climate (Parkash and Munjal 1999; Hoffmann et al. 2002; Arthur et al. 2008; Sinclair et al. 2012). However, these studies are recent (within the last 15 years), and at this stage cannot provide the temporal context. Temporal comparisons of data sets may also be problematic and require establishing a consistent and comparable measure of stress resistance that can easily translate into ecologically meaningful estimates to relate to climate (Chown et al. 2009; Rezende et al. 2011; Overgaard et al. 2012).

With a lack of extensive data sets to track responses to climate change, we focus on laboratory-based studies to examine the potential for species to respond via evolutionary or plastic processes. Using a common garden design, population comparisons in *Drosophila* have revealed clinal variation for stress traits, implying the presence of genetic variation. The relative role of climate in driving these patterns, however, is uncertain with clines in these traits tending to be inconsistent both across species and continents (Parkash and Munjal 2000; Hoffmann et al. 2001; Sarup et al. 2006; Arthur et al. 2008). Moreover, determining which traits are the direct targets of selection as well as the specific selection pressures remains elusive (Hoffmann and Weeks 2007). For other species, clinal comparisons of stress resistance traits are rare (Alford et al. 2012) and this in part may be due to the difficulty in rearing many species in a laboratory environment.

Laboratory-based quantitative genetic studies are generally rare, particularly outside of *Drosophila* species. This is simply due to the necessary scale of the experiments required to obtain accurate estimates of genetic variances and the difficulty in rearing many insects en masse in the laboratory. The largest comparison of estimates of evolutionary potential for climate-related traits to date is that for desiccation and cold resistance in five tropical and five temperate *Drosophila* species (Kellermann et al. 2009). In contrast to temperate species, low levels of genetic variation were found in all tropical species, suggesting low potential to increase their resistance to cold or dry environments. With desiccation resistance, in particular, emerging as an important trait in terms of climate change responses (Kearney et al. 2009a; Bonebrake and Mastrandrea 2010; Clusella-Trullas et al. 2011; Kellermann et al. 2012a), these results suggest tropical species may be constrained in their climate change responses. In other widespread insect species, heritable variation for desiccation resistance has also been detected (Sota 1993; Li and Heinz 1998; Kearney et al. 2009a).

For heat resistance, even fewer data exist. In *D. melanogaster*, estimates of genetic variances for heat tolerance can be low and selection experiments often rapidly reach plateaus, suggesting evolutionary responses may be limited (Gilchrist and Huey 1999; Mitchell and Hoffmann 2010; Hoffmann et al. 2012). A study encompassing estimates of upper thermal limits in ∼90 *Drosophila* species found that only a handful of species had evolved high heat tolerance, and these were restricted to two species groups when mapped onto a phylogeny (Kellermann et al. 2012b). This suggests that present day heat tolerances may be constrained by evolutionary history (Wiens et al. 2010). These examples in *Drosophila*, combined with patterns of low variance for upper thermal limits in insects (Addo-Bediako et al. 2000; Deutsch et al. 2008), suggest that evolutionary shifts in heat resistance may be slow. Yet in other terrestrial invertebrates, high heat resistance does not appear to be limited, with upper thermal limits of some ant species upwards of 50°C (Lighton and Turner 2004).

High levels of phenotypic plasticity, in stress resistance traits, have been documented for many species (Hoffmann et al. 2003; Angilletta 2009). Most studies have focused on cold-hardening (short-term exposures to rapid temperature changes) and acclimation (long-term exposures), demonstrating a high level of plasticity in cold resistance reviewed in MacMillan and Sinclair (2011). Fewer studies have considered plasticity in heat resistance (Bahrndorff et al. 2009; Fischer et al. 2010; Sobek et al. 2011) and even fewer in desiccation resistance (Hoffmann 1990, 1991; Bubliy et al. 2012). Here, plastic responses tend to be smaller than in cold resistance (Chown 2001; Overgaard et al. 2011) and dependent on how the trait is measured (Hoffmann et al. 2003). A comparative study of *Drosophila* species demonstrated little capacity for phenotypic plasticity in upper thermal limits (Overgaard et al. 2011), but when heat resistance was measured via an alternative method, plastic responses were detected (Hoffmann et al. 2003; Mitchell et al. 2011). Further work quantifying the potential for plastic responses in *Drosophila* and other taxa is needed.

### Symbionts

It is interesting to consider symbiotic relationships in the context of adaptive and plastic responses to climate change (see also paper on marine plants and animals in this Special Issue). On the one hand, symbionts are not strictly speaking a species trait of their host, but rather a very intimate ecological interaction. On the other hand, in some of these interactions, the symbiont resides in the cytoplasm (endosymbionts), is strictly vertically transmitted (hence, a genetic element) and coevolves with its host (O'Neill et al. 1997). We here mainly consider endosymbionts that are, to all intents and purposes, genetic traits of the host species, and, given the high mutation rate, any response to climatic changes is likely to be genetic. However, in symbionts that have a facultative, rather than obligate interaction with their host, the gain and loss of different symbionts strains may be seen as plastic, rather than adaptive.

Symbiotic relationships in insects may both facilitate and constrain evolutionary responses to rapidly changing environments (Dunbar et al. 2007; Gilbert et al. 2010; Wernegreen 2012). Insects and many other invertebrates quite commonly harbour a range of endosymbionts, which can be either obligate or facultative (Wernegreen 2012). Symbiotic relationships may facilitate adaptive processes by enabling species to exploit wider feeding niches (Feldhaar 2011), and a range of traits have been linked to symbionts, including pathogen resistance, reproductive manipulation and thermal tolerances (Montllor et al. 2002; Weeks et al. 2002b; Glaser and Meola 2010).

The potential for endosymbionts to alter the thermal tolerance of their hosts has long been recognized, with prolonged heat stress reducing, if not eliminating, the presence of many endosymbionts within their hosts (Ohtaka and Ishikawa 1991). For species not reliant on the symbiotic relationship, a reduction in fitness following a heat stress has been observed for temperatures between 25 and 28°C (Montllor et al. 2002). For species that harbour an obligate relationship, such as is the case for the endosymbiotic bacterium *Buchnera*, which supply essential nutrients to aphid species (Baumann 2005), the ecological tolerances of the endosymbionts will play a key role in driving species distributions and responses to climate change (Dunbar et al. 2007; Wernegreen 2012). *Buchnera* in particular are highly sensitive to heat stress and are likely to have limited potential for evolutionary responses (Dunbar et al. 2007). These symbionts have been linked to distributional limits in aphid species (Montllor et al. 2002; Chiu et al. 2012) and thus could represent a constraint in terms of species persistence under climate change scenarios.

Positive effects on thermal tolerance have also been demonstrated with the presence of a facultative (secondary) endosymbiont enhancing reproduction in the pea aphid (*Acyrthosiphon pisum*) following a heat stress (Montllor et al. 2002), directly increasing heat tolerance in the whitefly (Brumin et al. 2011) and in *A. pisum* (Chen et al. 2000; Russell and Moran 2006). Russell and Moran (2006) demonstrated that the presence of secondary endosymbionts increased the survival of bacteriocytes (*Buchnera* housing cells) following a heat stress, presumably increasing the survival of *Buchnera* and providing a possible mechanism for increased heat tolerance. The relationship between host and endosymbionts is clearly complex, with secondary endosymbionts likely to play a larger role in facilitating responses to climate change.

### Anonymous traits

Perhaps one of the best examples of a genetic response to climate change in insects is the temporal shift in clinal allele frequencies and inversion polymorphisms in *Drosophila* species (*D. melanogaster*: Anderson et al. 2005; Umina et al. 2005; *D. subobscura*: Balanya et al. 2009; *D. robusta*: Etges and Levitan 2008). These authors demonstrate a shift in allele and inversion frequencies coinciding with a shift in temperature over a 30- to 20-year period. In addition, short-term responses due to fluctuating thermal selection in *D. subobscura* have also been reported (Rodríguez-Trelles et al. 2013). With the presence of latitudinal clines in stress traits commonly found in *Drosophila*, it is tempting to link these observed shifts in gene and inversion frequency clines to these traits. For *D. melanogaster,* clines in stress traits have been found for cold, heat and desiccation stress (Parkash and Munjal 2000; Hoffmann et al. 2002), and genes inside the inversion have been tentatively linked to stress tolerance (Weeks et al. 2002a; Rego et al. 2010). In *D. subobscura*, different chromosomal arrangements appear linked to thermal tolerance, but no study has addressed whether thermal tolerance itself clines (Rego et al. 2010). Inversion polymorphisms may cline in *D. robusta*, but the phenotypic data are missing. Moreover, consistent patterns in clinal variation are not always found (Parkash and Munjal 2000; Hoffmann et al. 2001; Griffiths et al. 2005; Sarup et al. 2006; Arthur et al. 2008), highlighting the complexity of clinal trait evolution. Trait variation is likely to be shaped by a range of abiotic and biotic interactions, which themselves may also cline with latitude. Thus, whether temperature is the main driver of clinal variation remains to be determined.

## Discussion

Many invertebrates are arguably easier to work with under experimental conditions both in the laboratory and field than vertebrates and therefore should make ideal subjects for proving either evolutionary adaptation or phenotypic plasticity under climate change. Yet, even in this group of organisms, as our review shows, few convincing cases for either have been reported. We divided our review of the literature into the following five sections: (i) changes in the way an organism deals behaviourally and ecologically with its external environment after climate change, (ii) Changes in the interface between an organism's internal and external environment (i.e. in the colour of its skin or carapace) after climate change, (iii) Changes in the way an organism deals physiologically with its internal environment after climate change, (iv) Changes that take place in a species' endosymbionts, rather than in the host itself, and Finally, we also briefly considered (v) conspicuous, climate-change-associated changes in ‘anonymous’ genetic markers for which the function is unknown.

To begin with the last of these, the genetic basis for allele and inversion polymorphisms in *Drosophila* is, by definition, undisputed. However, as only correlative evidence exists, a firm conclusion of adaptive evolution under the influence of climate change cannot be drawn, although it appears likely that many of these markers are associated with genes that affect heat tolerance and other climate-related characteristics. Similarly, tenuous are the changes in a species endosymbionts. Temperature stress may destroy endosymbiotic bacteria, with effects on the host's metabolism (in the case of, e.g. *Buchnera* in aphids) or reproduction (*Wolbachia* in certain insects), but the presence or absence of endosymbionts itself is not easily classed as either genetic or plastic. However, given the short generation time of these symbionts, adaptation is a likely possibility. In fact, we suggest more attention is paid to the role of symbionts in temperature stress responses in invertebrates in general (see also Reusch et al. 2014).

Given the fact that integumental colour in invertebrates and its role in mediating body temperature have been a focus for population and ecological genetics for almost a century, it is not surprising that a large amount of data exist for these trait systems. The evidence for a genetic basis of these traits is usually good, although it is important to note that the impact of phenotypic plasticity varies greatly, even among closely related species, whereas shell colour in *Cepaea* is almost entirely hereditary, in the related helicid snail *Theba*, there appears to be a certain degree of phenotypic plasticity. Similarly, the coccinellids *Adalia* and *Harmonia* differ in the size of the genetic component in the melanization of their elytra. Therefore, although the bulk of data suggest that body colour change under temperature change is firmly associated with evolutionary adaptation, we suggest there is a risk of ignoring the alternative of phenotypic plasticity. We would therefore suggest that the heritability of the colour trait is measured in each individual species and not to extrapolate from related species. Moreover, the impact body colour (and the concomitant change in body temperature) has on behaviour is also often ignored. The work in *Cepaea* and *Tetrix* suggests that complex inter-relations exist and that the tradition of studying colour polymorphism in isolation from behavioural traits should be abandoned.

Behavioural changes under temperature change are surprisingly varied. These include microhabitat choice, as well as greater rates of dispersal at the leading edge of an expanding biogeographical range. The latter also selects for dispersal-related traits such as wing length and investment in flight muscles. The evidence that behavioural changes are adaptive is relatively good, although there are complex links with integumental traits, such as the curious genetic complexes of behaviour and colour in pygmy grasshoppers (Forsman et al. 2002). Similar situations are likely to exist in other systems as well.

Physiological and life-history traits are arguably the most important in the context of climate change. For life-history traits, extensive temporal data sets exist, yet most studies focus on correlative patterns between traits and climate rather than examining what drives the observed the patterns. For physiological stress traits, temporal data sets simply do not exist, and the data that do exist are strongly focused on *Drosophila* only. Overall, depending on the species, both genetic and plastic responses exist in phenology and other life-history traits and these traits are changing in response to climate. However, experiments explicitly testing for genetic and plastic responses are needed. Data from laboratory-based studies suggest the potential for evolutionary responses for stress traits desiccation and cold resistance for widespread species, while an absence of genetic variance in tropical species suggests constraints to evolutionary change. Although generally, these traits appear highly plastic, more data contrasting plastic responses in restricted versus widespread species are needed. Emerging trends suggest heat resistance may be evolutionarily constrained generally across species, and more work is needed to confirm if this pattern is general. Phenotypic plasticity in heat resistance may also be limited, but once again more work is needed, particularly on a broad range of taxa. The challenge is to extend quantitative genetic studies to non-*Drosophila* systems but also to move from laboratory to field-based estimates of evolutionary potential.

## References

[b1] Addo-Bediako A, Chown SL, Gaston KJ (2000). Thermal tolerance, climatic variability and latitude. Proceedings of the Royal Society of London B: Biological Sciences.

[b2] Alford L, Blackburn TM, Bale JS (2012). Effect of latitude and acclimation on the lethal temperatures of the peach-potato aphid *Myzus persicae*. Agricultural and Forest Entomology.

[b3] Altermatt F (2010). Climatic warming increases voltinism in European butterflies and moths. Proceedings of the Royal Society of London B: Biological Sciences.

[b4] Anderson AR, Hoffmann AA, McKechnie SW, Umina PA, Weeks AR (2005). The latitudinal cline in the In(3R)Payne inversion polymorphism has shifted in the last 20 years in Australian *Drosophila melanogaster* populations. Molecular Ecology.

[b5] Angilletta MJ (2009). Thermal adaptation: A theoretical and empirical synthesis.

[b6] Arthur AL, Weeks AR, Sgro CM (2008). Investigating latitudinal clines for life history and stress resistance traits in *Drosophila simulans* from eastern Australia. Journal of Evolutionary Biology.

[b7] Bahrndorff S, Maien J, Loeschcke V, Ellers J (2009). Dynamics of heat-induced thermal stress resistance and Hsp70 expression in the springtail, *Orchesella cincta*. Functional Ecology.

[b145] Balanya J, Oller JM, Huey RB, Gilchrist GW, Serra L (2006). Global genetic change tracks global climate warming in Drosophila subobscura. Science.

[b8] Balanya J, Huey RB, Gilchrist GW, Serra L (2009). The chromosomal polymorphism of *Drosophila subobscura*: a microevolutionary weapon to monitor global change. Heredity.

[b9] Baumann P (2005). Biology of bacteriocyte-associated endosymbionts of plant sap-sucking insects. Annual Review of Microbiology.

[b10] Bentz BJ, Bracewell RR, Mock KE, Pfrender ME (2011). Genetic architecture and phenotypic plasticity of thermally-regulated traits in an eruptive species, *Dendroctonus ponderosae*. Evolutionary Ecology.

[b11] Bonebrake TC, Mastrandrea MD (2010). Tolerance adaptation and precipitation changes complicate latitudinal patterns of climate change impacts. Proceedings of the National Academy of Sciences of the United States of America.

[b12] Bradford MJ, Roff DA (1995). Genetic and phenotypic sources of life-history variation along a cline in voltinism in the cricket *Allonemobius socius*. Oecologia.

[b13] Bradshaw WE, Holzapfel CM (2001). Genetic shift in photoperiodic response correlated with global warming. Proceedings of the National Academy of Sciences of the United States of America.

[b14] Brakefield PM (1987). Industrial melanism: do we have the answers?. Trends in Ecology and Evolution.

[b15] Brakefield PM, de Jong PW (2011). A steep cline in ladybird melanism has decayed over 25 years: a genetic response to climate change?. Heredity.

[b16] Brakefield PM, Willmer PG (1985). The basis of thermal melanism in the ladybird *Adalia bipunctata* – differences in reflectance and thermal-properties between the morphs. Heredity.

[b17] Brumin M, Kontsedalov S, Ghanim M (2011). *Rickettsia* influences thermotolerance in the whitefly *Bemisia tabaci* B biotype. Insect Science.

[b18] Bubliy OA, Kristensen TN, Kellermann V, Loeschcke V (2012). Plastic responses to four environmental stresses and cross-resistance in a laboratory population of *Drosophila melanogaster*. Functional Ecology.

[b19] Cain AJ, Russell-Hunter WD (1983). Ecology and ecogenetics of terrestrial molluscan populations. The Mollusca, volume 6, Ecology.

[b20] Cain AJ, Sheppard PM (1950). Selection in the polymorphic land snail *Cepaea nemoralis*. Heredity.

[b21] Cain AJ, King JMB, Sheppard PM (1960). New data on the genetics of polymorphism in the snail *Cepaea nemoralis* L. Genetics.

[b22] Cameron RAD, Cook LM (2013). Temporal morph frequency changes in sand-dune populations of *Cepaea nemoralis* (L.). Biological Journal of the Linnean Society.

[b23] Cameron RAD, Pokryszko BM (2008). Variation in *Cepaea* populations over 42 years: climate fluctuations destroy a topographical relationship of morph-frequencies. Biological Journal of the Linnean Society.

[b24] Cameron RAD, Cook LM, Greenwood JJD (2013). Change and stability in a steep morph-frequency cline in the snail *Cepaea nemoralis* (L.) over 43 years. Biological Journal of the Linnean Society.

[b25] Chen DQ, Montllor CB, Purcell AH (2000). Fitness effects of two facultative endosymbiotic bacteria on the pea aphid, *Acyrthosiphon pisum*, and the blue alfalfa aphid, A-kondoi. Entomologia Experimentalis et Applicata.

[b26] Chiu MC, Chen YH, Kuo MH (2012). The effect of experimental warming on a low-latitude aphid, *Myzus varians*. Entomologia Experimentalis et Applicata.

[b27] Chown SL (2001). Physiological variation in insects: hierarchical levels and implications. Journal of Insect Physiology.

[b28] Chown SL, Jumbam KR, Srensen JG, Terblanche JS (2009). Phenotypic variance, plasticity and heritability estimates of critical thermal limits depend on methodological context. Functional Ecology.

[b29] Clusella-Trullas S, Blackburn TM, Chown SL (2011). Climatic predictors of temperature performance curve parameters in ectotherms imply complex responses to climate change. The American Naturalist.

[b30] Cook LM (1967). The genetics of *Cepaea nemoralis*. Heredity.

[b31] Cowie RH (1984). Ecogenetics of *Theba pisana* (Pulmonata: Helicidae) at the northern edge of its range. Malacologia.

[b32] Cowie RH (1990). Climatic selection on body colour in the land snail *Theba pisana* (Pulmonata: Helicidae). Heredity.

[b33] Cowie RH, Jones JS (1985). Climatic selection on body colour in *Cepaea*. Heredity.

[b34] Deutsch CA, Tewksbury JJ, Huey RB, Sheldon KS, Ghalambor CK, Haak DC, Martin PR (2008). Impacts of climate warming on terrestrial ectotherms across latitude. Proceedings of the National Academy of Sciences of the United States of America.

[b35] Dingemanse NJ, Kalkman VJ (2008). Changing temperature regimes have advanced the phenology of Odonata in the Netherlands. Ecological Entomology.

[b36] Doi H (2008). Delayed phenological timing of dragonfly emergence in Japan over five decades. Biology Letters.

[b37] Donnelly A, Caffarra A, Kelleher CT, O'Neill BF, Diskin E, Pletsers A, Proctor H (2012). Surviving in a warmer world: environmental and genetic responses. Climate Research.

[b38] Dunbar HE, Wilson ACC, Ferguson NR, Moran NA (2007). Aphid thermal tolerance is governed by a point mutation in bacterial symbionts. PLoS Biology.

[b39] Ellwood ER, Diez JM, Ibanez I, Primack RB, Kobori H, Higuchi H, Silander JA (2012). Disentangling the paradox of insect phenology: are temporal trends reflecting the response to warming?. Oecologia.

[b40] Endler JA (1986). Natural Selection in the Wild.

[b41] Etges WJ, Levitan M (2008). Variable evolutionary response to regional climate change in a polymorphic species. Biological Journal of the Linnean Society.

[b42] Feldhaar H (2011). Bacterial symbionts as mediators of ecologically important traits of insect hosts. Ecological Entomology.

[b43] Fischer K, Dierks A, Franke K, Geister TL, Liszka M, Winter S, Pflicke C (2010). Environmental effects on temperature stress resistance in the tropical butterfly *Bicyclus anynana*. PLoS ONE.

[b44] Forsman A, Ringblom K, Civantos E, Ahnesjö J (2002). Coevolution of color pattern and thermoregulatory behavior in polymorphic pygmy grasshoppers *Tetrix undulata*. Evolution.

[b45] Gibbs AG, Perkins MC, Markow TA (2003). No place to hide: microclimates of Sonoran desert *Drosophila*. Journal of Thermal Biology.

[b46] Gienapp P, Teplitsky C, Alho JS, Mills JH, Merilä J (2008). Climate change and evolution: disentangling environmental and genetic responses. Molecular Ecology.

[b47] Gilbert SF, McDonald E, Boyle N, Buttino N, Gyi L, Mai M, Prakash N (2010). Symbiosis as a source of selectable epigenetic variation: taking the heat for the big guy. Philosophical Transactions of the Royal Society B: Biological Sciences.

[b48] Gilchrist GW, Huey RB (1999). The direct response of *Drosophila melanogaster* to selection on knockdown temperature. Heredity.

[b49] Glaser RL, Meola MA (2010). The native *Wolbachia* endosymbionts of *Drosophila melanogaster* and *Culex quinquefasciatus* increase host resistance to west nile virus infection. PLoS ONE.

[b50] Griffiths JA, Schiffer M, Hoffmann AA (2005). Clinal variation and laboratory adaptation in the rainforest species *Drosophila birchii* for stress resistance, wing size, wing shape and development time. Journal of Evolutionary Biology.

[b51] Harrington R, Clark SJ, Welham SJ, Verrier PJ, Denholm CH, Hulle M, Maurice D (2007). Environmental change and the phenology of European aphids. Global Change Biology.

[b52] Hassall C, Thompson DJ, French GC, Harvey IF (2007). Historical changes in the phenology of British Odonata are related to climate. Global Change Biology.

[b155] Heath DJ (1975). Colour, sunlight and internal temperatures in the land-snail Cepaea nemoralis. Oecologia.

[b53] Hill JK, Thomas CD, Lewis OT (1999). Flight morphology in fragmented populations of a rare British butterfly, *Hesperia comma*. Biological Conservation.

[b54] Hoffmann AA (1990). Acclimation for desiccation resistance in *Drosophila melanogaster* and the assocation between acclimation responses and genetic variation. Journal of Insect Physiology.

[b55] Hoffmann AA (1991). Acclimation for desiccation resistance in *Drosophila* species and population comparisons. Journal of Insect Physiology.

[b56] Hoffmann AA, Weeks AR (2007). Climatic selection on genes and traits after a 100 year-old invasion: a critical look at the temperate-tropical clines in *Drosophila melanogaster* from eastern Australia. Genetica.

[b57] Hoffmann AA, Hallas R, Sinclair C, Mitrovski P (2001). Levels of variation in stress resistance in *Drosophila* among strains, local populations, and geographic regions: patterns for desiccation, starvation, cold resistance, and associated traits. Evolution.

[b58] Hoffmann AA, Anderson A, Hallas R (2002). Opposing clines for high and low temperature resistance in *Drosophila melanogaster*. Ecology Letters.

[b59] Hoffmann AA, Sorensen JG, Loeschcke V (2003). Adaptation of *Drosophila* to temperature extremes: bringing together quantitative and molecular approaches. Journal of Thermal Biology.

[b60] Hoffmann AA, Chown SL, Clusella-Trullas S (2012). Upper thermal limits in terrestrial ectotherms: how constrained are they?. Functional Ecology.

[b61] Huey RB, Deutsch CA, Tewksbury JJ, Vitt LJ, Hertz PE, Perez HJA, Garland T (2009). Why tropical forest lizards are vulnerable to climate warming. Proceedings of the Royal Society of London B.

[b62] Huey RB, Kearney MR, Krockenberger A, Holtum JAM, Jess M, Williams SE (2012). Predicting organismal vulnerability to climate warming: roles of behaviour, physiology and adaptation. Philosophical Transactions of the Royal Society B: Biological Sciences.

[b63] Husby A, Nussey DH, Visser ME, Wilson AJ, Sheldon BC, Kruuk LEB (2010). Contrasting patterns of phenotypic plasticity in reproductive traits in two great tit (*Parus major*) populations. Evolution.

[b64] Johnson MS (1980). Association of shell banding and habitat in a colony of the land snail *Theba pisana*. Heredity.

[b65] Johnson MS (2011). Thirty-four years of climatic selection in the land snail *Theba pisana*. Heredity.

[b66] Johnson MS (2012). Epistasis, phenotypic disequilibrium and contrasting associations with climate in the land snail *Theba pisana*. Heredity.

[b67] Jones JS (1982). Genetic differences in individual behaviour associated with shell polymorphism in the snail *Cepaea nemoralis*. Nature.

[b68] Jones JS, Leith BH, Rawlings P (1977). Polymorphism in *Cepaea*: a problem with too many solutions?. Annual Review of Ecology and Systematics.

[b69] de Jong PW, Gussekloo SWS, Brakefield PM (1996). Differences in thermal balance, body temperature and activity between non-melanic and melanic two-spot ladybird beetles (*Adalia bipunctata*) under controlled conditions. Journal of Experimental Biology.

[b70] Karl I, Janowitz SA, Fischer K (2008). Altitudinal life-history variation and thermal adaptation in the copper butterfly *Lycaena tityrus*. Oikos.

[b71] Karlsson M, Forsman A (2010). Is melanism in pygmy grasshoppers induced by crowding?. Evolutionary Ecology.

[b72] Kavaliers M (1992). Opioid systems, behavioral thermoregulation and shell polymorphism in the land snail, *Cepaea nemoralis*. Journal of Comparative Physiology.

[b73] Kearney M, Porter WP, Williams C, Ritchie S, Hoffmann AA (2009a). Integrating biophysical models and evolutionary theory to predict climatic impacts on species' ranges: the dengue mosquito *Aedes aegypti* in Australia. Functional Ecology.

[b74] Kearney M, Shine R, Porter WP (2009b). The potential for behavioral thermoregulation to buffer “cold-blooded” animals against climate warming. Proceedings of the National Academy of Sciences of the United States of America.

[b75] Kellermann V, Sgro B, van Heerwaarden CM, Hoffmann AA (2009). Fundamental evolutionary limits in ecological traits drive *Drosophila* species distributions. Science.

[b76] Kellermann V, Loeschcke V, Hoffmann AA, Kristensen TN, Fløjgaard C, David JR, Overgaard J (2012a). Phylogenetic constraints in key functional traits behind species' climate niches: patterns of desiccation and cold resistance across 95 *Drosophila* species. Evolution.

[b77] Kellermann V, Overgaard J, Hoffmann AA, Flojgaard C, Svenning JC, Loeschcke V (2012b). Upper thermal limits of *Drosophila* are linked to species distributions and strongly constrained phylogenetically. Proceedings of the National Academy of Sciences of the United States of America.

[b78] Köhler HR, Lazzara R, Dittbrenner N, Capowiez Y, Mazzia C, Tiebskorn R (2009). Shell phenotypic variation and stress proteins: do different heat response strategies contribute to Waddington's widget in field populations?. Journal of Experimental Zoology B: Molecular and Developmental Evolution.

[b79] Köhler HR, Schultz C, Scheil AE, Triebskorn R, Seifan M, Di Lellis MA (2013). Historic data analysis reveals ambient temperature as a source of phenotypic variation in populations of the land snail *Theba pisana*. Biological Journal of the Linnean Society.

[b80] Kopp M, Hendry AP (2014). Rapid evolution of quantitative traits: theoretical perspectives. Evolutionary Applications.

[b81] Lang A (1904). Über Vorversuche zu Untersuchungen über die Varietätsbildung von *Helix hortensis* Müller und *Helix nemoralis* L. Denkschriften des Medizinisch-Naturwissenschaftlichen Gesellschafts Jena.

[b82] Li JB, Heinz KM (1998). Genetic variation in desiccation resistance and adaptability of the red imported fire ant (Hymenoptera: Formicidae) to arid regions. Annals of the Entomological Society of America.

[b83] Lighton JRB, Turner RJ (2004). Thermolimit respirometry: an objective assessment of critical thermal maxima in two sympatric desert harvester ants, *Pogonomyrmex rugosus* and *P. californicus*. Journal of Experimental Biology.

[b84] MacArthur RH (1972). Geographical ecology.

[b85] MacMillan HA, Sinclair BJ (2011). Mechanisms underlying insect chill-coma. Journal of Insect Physiology.

[b86] Majerus MEN (1994). Ladybirds.

[b87] Majerus MEN (1998). Melanism: Evolution in Action.

[b88] Martin-Vertedor D, Ferrero-Garcia JJ, Torres-Vila LM (2010). Global warming affects phenology and voltinism of *Lobesia botrana* in Spain. Agricultural and Forest Entomology.

[b89] Mayr E (1963). Animal species and evolution.

[b90] Merilä J, Hendry A (2014). Climate change, adaptation, and phenotypic plasticity: the problem and the evidence. Evolutionary Applications.

[b91] Mitchell KA, Hoffmann AA (2010). Thermal ramping rate influences evolutionary potential and species differences for upper thermal limits in *Drosophila*. Functional Ecology.

[b92] Mitchell KA, Sgro CM, Hoffmann AA (2011). Phenotypic plasticity in upper thermal limits is weakly related to *Drosophila* species distributions. Functional Ecology.

[b93] Mitchie LJ, Mallard F, Majerus MEN, Jiggins FM (2010). Melanic through nature or nurture: genetic polymorphism and phenotypic plasticity in *Harmonia axyridis*. Journal of Evolutionary Biology.

[b94] Montllor CB, Maxmen A, Purcell AH (2002). Facultative bacterial endosymbionts benefit pea aphids *Acyrthosiphon pisum* under heat stress. Ecological Entomology.

[b95] Munjal AK, Karan D, Gibert P, Moreteau B, Parkash R, David JR (1997). Thoracic trident pigmentation in *Drosophila melanogaster*: latitudinal and altitudinal clines in Indian populations. Genetics Selection Evolution.

[b96] Murray J, King RC (1975). The Genetics of the Mollusca. Handbook of Genetics.

[b97] Nylin S, Gotthard K (1998). Plasticity in life-history traits. Annual Review of Entomology.

[b98] Ohtaka C, Ishikawa H (1991). Effects of heat treatment on the symbiotic system of an aphid mycetocyte. Symbiosis.

[b99] O'Neill SL, Hoffmann AA, Werren JH (1997). Influential Passengers: Inherited Microorganisms and Arthropod Reproduction.

[b100] Overgaard J, Kristensen TN, Mitchell KA, Hoffmann AA (2011). Thermal tolerance in widespread and tropical *Drosophila* species: does phenotypic plasticity increase with latitude?. American Naturalist.

[b101] Overgaard J, Kristensen TN, Sorensen JG (2012). Validity of the thermal ramping assays used to assess thermal tolerance in arthropods. PLoS ONE.

[b102] Ozgo M (2008). Current problems in the research of *Cepaea* polymorphism. Folia Malacologica.

[b103] Ozgo M (2011). Rapid evolution in unstable habitats: a success story of the polymorphic land snail *Cepaea nemoralis* (Gastropoda: Pulmonata). Biological Journal of the Linnean Society.

[b104] Ozgo M, Bogucki Z (2011). Colonization, stability, and adaptation in a transplant experiment of the polymorphic land snail *Cepaea nemoralis* (Gastropoda: Pulmonata) at the edge of its geographical range. Biological Journal of the Linnean Society.

[b105] Ozgo M, Kinnison MT (2008). Contingency and determinism during convergent contemporary evolution in the polymorphic land snail, *Cepaea nemoralis*. Evolutionary Ecology Research.

[b106] Ozgo M, Kubea A (2005). Humidity and the effect of shell colour on activity of *Cepaea nemoralis* (Linnaeus, 1758). Folia Malacologica.

[b157] Ozgo M, Schilthuizen M (2012). Evolutionary change in Cepaea nemoralis shell colour over 43 years. Global Change Biology.

[b107] Parkash R, Munjal AK (1999). Climatic selection of starvation and desiccation resistance in populations of some tropical drosophilids. Journal of Zoological Systematics and Evolutionary Research.

[b108] Parkash R, Munjal AK (2000). Evidence of independent climatic selection for desiccation and starvation tolerance in Indian tropical populations of *Drosophila melanogaster*. Evolutionary Ecology Research.

[b109] Poyry J, Leinonen R, Soderman G, Nieminen M, Heikkinen RK, Carter TR (2011). Climate-induced increase of moth multivoltinism in boreal regions. Global Ecology and Biogeography.

[b110] Rego C, Balanya J, Fragata I, Matos M, Rezende EL, Santos M (2010). Clinal patterns of chromosomal inversion polymorphisms in *Drosophila subobscura* are partly associated with the thermal preferences and heat stress resistance. Evolution.

[b111] Reusch TBH (2014). Climate change in the oceans: evolutionary vs. phenotypically plastic responses of marine animals and plants. Evolutionary Applications.

[b112] Rezende EL, Tejedo M, Santos M (2011). Estimating the adaptive potential of critical thermal limits: methodological problems and evolutionary implications. Functional Ecology.

[b113] Richardson AMM (1974). Differential climatic selection in natural populations land snail *Cepaea nemoralis*. Nature.

[b114] Rodríguez-Trelles F, Tarrío R, Santos M (2013). Genome-wide evolutionary response to a heat-wave in *Drosophila*. Biology Letters.

[b115] Roy DB, Sparks TH (2000). Phenology of British butterflies and climate change. Global Change Biology.

[b116] Russell JA, Moran NA (2006). Costs and benefits of symbiont infection in aphids: variation among symbionts and across temperatures. Proceedings of the Royal Society B: Biological Sciences.

[b117] Sarup P, Sorensen JG, Dimitrov K, Barker JSF, Loeschcke V (2006). Climatic adaptation of *Drosophila buzzatii* populations in southeast Australia. Heredity.

[b118] Scheil AE, Gärtner U, Köhler HR (2012). Colour polymorphism and thermal capacities in *Theba pisana* (O. F. Müller 1774). Journal of Thermal Biology.

[b119] Schilthuizen M (2013). Rapid, habitat related evolution of land snail colour morphs on reclaimed land. Heredity.

[b120] Silvertown J, Cook L, Cameron R, Dodd M, McConway K, Worthington J, Skelton P (2011). Citizen science reveals unexpected continental-scale evolutionary change in a model organism. PLoS ONE.

[b121] Sinclair BJ, Williams CM, Terblanche JS (2012). Variation in thermal performance among insect populations. Physiological and Biochemical Zoology.

[b122] Sobek S, Rajamohan A, Dillon D, Cumming RC, Sinclair BJ (2011). High temperature tolerance and thermal plasticity in emerald ash borer *Agrilus planipennis*. Agricultural and Forest Entomology.

[b123] Sota T (1993). Response to selection for desiccation resistance in *Aedes albopictus* eggs (Diptera, Culicidae). Applied Entomology and Zoology.

[b124] Sparks TH, Langowska A, Glazaczow A, Wilkaniec Z, Bienkowska M, Tryjanowski P (2010). Advances in the timing of spring cleaning by the honeybee *Apis mellifera* in Poland. Ecological Entomology.

[b156] Steigen AL (1979). Temperature effects on energy metabolism in banded and unbanded morphs of the snail Cepaea hortensis Mull. Oecologia.

[b125] Sunday JM, Bates AE, Dulvy NK (2011). Global analysis of thermal tolerance and latitude in ectotherms. Proceedings of the Royal Society of London B: Biological Sciences.

[b126] Tauber MJ, Tauber CA, Masaki S (1986). Seasonal Adaptation of Insects.

[b127] Telonis-Scott M, Hoffmann AA, Sgro CM (2011). The molecular genetics of clinal variation: a case study of ebony and thoracic trident pigmentation in *Drosophila melanogaster* from eastern Australia. Molecular Ecology.

[b128] Thomas CD, Bodsworth EJ, Wilson RJ, Simmons AD, Davies ZG, Musche M, Conradt L (2001). Ecological and evolutionary processes at expanding range margins. Nature.

[b129] Trullas SC, Spotila JH, van Wyk JR (2007). Thermal melanism in ectotherms. Journal of Thermal Biology.

[b130] Umina PA, Weeks AR, Kearney MR, McKechnie SW, Hoffmann AA (2005). A rapid shift in a classic clinal pattern in *Drosophila* reflecting climate change. Science.

[b131] Valimaki P, Kivela SM, Maenpaa MI, Tammaru T (2013). Latitudinal clines in alternative life histories in a geometrid moth. Journal of Evolutionary Biology.

[b132] Weeks AR, McKechnie SW, Hoffmann AA (2002a). Dissecting adaptive clinal variation: markers, inversions and size/stress associations in *Drosophila melanogaster* from a central field population. Ecology Letters.

[b133] Weeks AR, Reynolds KT, Hoffmann AA, Mann H (2002b). *Wolbachia* dynamics and host effects: what has (and has not) been demonstrated?. Trends in Ecology and Evolution.

[b134] Wernegreen JJ (2012). Mutualism meltdown in insects: bacteria constrain thermal adaptation. Current Opinion in Microbiology.

[b135] West-Eberhard MJ (2003). Developmental Plasticity and Evolution.

[b136] Westwood AR, Blair D (2010). Effect of regional climate warming on the phenology of butterflies in boreal forests in Manitoba, Canada. Environmental Entomology.

[b137] Wiens JJ, Ackerly DD, Allen AP, Anacker BL, Buckley LB, Cornell HV, Damschen EI (2010). Niche conservatism as an emerging principle in ecology and conservation biology. Ecology Letters.

[b138] Wittkopp PJ, Beldade P (2009). Development and evolution of insect pigmentation: genetic mechanisms and the potential consequences of pleiotropy. Seminars in Cell and Developmental Biology.

[b139] Wittkopp PJ, Stewart EE, Arnold LL, Neidert AH, Haerum BK, Thompson EM, Akhras S (2009). Intraspecific polymorphism to interspecific divergence: genetics of pigmentation in *Drosophila*. Science.

[b140] Wolda H (1965). Some preliminary observations on the distribution of the various morphs within natural populations of the polymorphic landsnail *Cepaea nemoralis* (L.). Archives Néerlandaises de Zoologie.

[b141] Zera AJ, Denno RF (1997). Physiology and ecology of dispersal polymorphisms in insects. Annual Review of Entomology.

[b142] Zhou XL, Harrington R, Woiwod IP, Perry JN, Bale JS, Clark SJ (1995). Effects of temperature on aphid phenology. Global Change Biology.

